# PPAR Ligands for Cancer Chemoprevention

**DOI:** 10.1155/2008/548919

**Published:** 2008-05-11

**Authors:** Yumiko Yasui, Mihye Kim, Takuji Tanaka

**Affiliations:** Department of Oncologic Pathology, Kanazawa Medical University, 1-1 Daigaku, Uchinada, Ishikawa 920-0293, Japan

## Abstract

Peroxisome proliferators-activated receptors (PPARs) that are members of the nuclear receptor superfamily have three different isoforms: PPAR*α*, PPAR*δ*, and PPAR*γ*. PPARs are ligand-activated transcription factors, and they are implicated in tumor progression, differentiation, and apoptosis. Activation of PPAR isoforms lead to both anticarcinogenesis and anti-inflammatory effect. It has so far identified many PPAR ligands including chemical composition and natural occurring. PPAR ligands are reported to activate PPAR signaling and exert cancer prevention and treatment in vitro and/or in vivo studies. Although the effects depend on the isoforms and the types of ligands, biological modulatory activities of PPARs in carcinogenesis and disease progression are attracted for control or combat cancer development. This short review summarizes currently available data on the role of PPAR ligands in carcinogenesis.

## 1. INTRODUCTION

**Figure 1:**
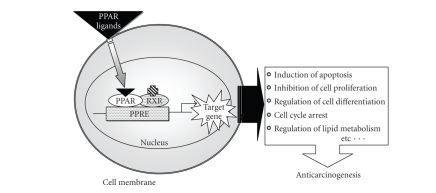
PPAR activation pathway and its target genes.

Peroxisome proliferators-activated receptors (PPARs) are member of the nuclear hormone receptor superfamily that were initially characterized as molecules that mediated the proliferation of peroxisomes in rodent liver parenchymal cells in response to the hypolipidemic drug clofibrate [1]. Subsequently, PPARs have been shown to regulate the expression of genes involved in a variety of biological processes, including lipid metabolism and insulin sensitivity [2, 3]. Three isotypes of PPAR exist; PPAR*α*, PPAB*β*/*δ* or simply *δ*, and PPAR*γ* which are known and they are encoded by three separate genes and display distinctly different tissue distributions and functions. PPAR*α* regulates numerous aspects of fatty acid catabolism, where as PPAR*γ* controls adipocyte differentiation, systemic glucose levels, and lipid homeostasis [4, 5]. PPAR*δ* is involved in development, embryo implantation, myelination of the corpus callosum, lipid metabolism, and epidermal cell proliferation [6]. The PPARs are ligand-dependent transcription factors that regulate target genes expression by binding to characteristic DNA sequences termed peroxisome proliferators response element (PPREs) located in the 5′ -flanking region of target genes [7, 8]. Each receptor binds to its PPRE as a heterodimer with the receptor for 9-cis retinoic acid, the retinoid X receptor (RXR) (Figure 1). Upon binding a ligand, the conformation of a PPAR is altered and stabilized such that a binding cleft is created, and recruitment of transcriptional coactivators occurs. The result is an increase in gene transcription, therefore PPARs are able to regulate such divers effects as cell proliferation, differentiation, or apoptosis.

## 2. PPAR*α* LIGANDS AND CARCINOGENESIS

PPAR*α* is the first member of this nuclear receptor subclass to be cloned [9]. PPAR*α* is expressed preferentially in the liver [10] and tissues with high fatty acid catabolism, such as the kidney, heart, skeletal muscle, and brown fat [11–13]. The PPAR*α* isotype is the cellular target for leukotriene B4 (LTB4) fibrates such as bezafibrate and fenofibrate, which are hypolipidemic drugs widely used for reducing triglyceride levels, a risk of cardiovascular diseases. Several studies have established a link between PPAR*α* activation and epidermal differentiation. Fibrates induce differentiation and inhibit proliferation in normal and hyperproliferating mouse epidermis and regulate apoptosis, but are inactive in PPAR*α*-deficient mice [14, 15]. Farnesol also stimulates PPAR*α*-dependent differentiation in epidermal keratinocytes [16]. Topical PPAR*α* ligands have weak preventive effects on tumor promotion in mouse skin, despite upregulation of PPAR*α* in untreated tumors compared with normal epidermis [17]. These observations suggest that the use of PPAR*α* activators may have chemopreventive properties in skin carcinogenesis. PPAR*α* expression is also upregulated in human prostate adenocarcinomas [18]. In addition, PPAR*α* ligands suppress the growth of several cancer lines, including colon [19], endometrial [20], and breast [21] in vivo or in vitro. PPAR*α* ligands are able to suppress the metastatic potential of melanoma cells in vivo and in vitro [22, 23]. More recently, a PPAR*α* ligand WY14643 suppresses both endothelial cell proliferation and tumorigenesis in a PPAR*α*-dependent manner [24]. These data suggest that certain PPAR*α* ligands may act as antitumor agents, although the exact mechanisms remain unclear. PPAR*α* activation has been associated with both anti and proinflammatory actions in rodents. PPAR*α* ligands reduce expression of inflammatory markers [25]. In contrast, the expression of the inflammatory mediator cyclooxygenase (COX)-2 in human breast and colon cancer cells is upregulated by PPAR*α* ligands [26]. The increased COX-2 expression is known to link to the risk of epithelial malignancies [27]. These findings indicate that PPAR*α* ligands may be interesting candidates for the chemoprevention of several types of cancers, but we should consider negative face of influence of PPAR*α* ligands on cancer development.

## 3. PPAR*δ* LIGANDS AND CARCINOGENESIS

A number of reports have described a variety of biological functions of the PPAR*α* and *γ* isotypes. These two isotypes also have clinical significance in the treatment of dyslipideamia and type II diabetes mellitus [28]. In contrast, less is known about the physiological role of the PPAR*δ* isoform, although there is some evidence supporting its involvement in embryo implantation and development [6, 29], epidermal maturation and wound healing [30], and regulation of fatty acid metabolism [31]. Recently, the effect of PPAR*δ* function on colon carcinogenesis has been reported. However, the role of PPAR*δ* in colon cancer is still unclear, as there are data suggesting that it either inhibits or promotes colon carcinogenesis. PPAR*δ* expression is increased in colon tumor cells with a mutant *Apc (adenomatous polyposis coli)* allele (min) [32]. The number of polyps was the same among the multiple intestinal neoplasia (Min) mice that were *Ppard* ^−/−^, *Ppard* ^+/−^, or *Ppard* ^+/+^. These findings suggest that PPAR*δ* is not essential for colon carcinogenesis, but PPAR*δ* may affect size and/or growth of polyps [29]. The most striking results were provided by a study demonstrating that in PPAR*δ* deficient (*Ppard^−/−^*) mice, both *Min* mutants and those with chemically induced cancers, colon polyp formation was significantly greater in those nullizygous for PPAR*δ* [33]. These results suggest that PPAR*δ* attenuates colon carcinogenesis. On the other hand, the following observations strongly suggest that PPAR*δ* enhances colon cancer formation. PPAR*δ* was elevated in colon cancer cells and was repressed by *APC* gene via the *β*-catenin/Tcf-4 response elements in its promoter [32]. Genetic disruption of PPAR*δ* decreases the tumorigenicity of human colon cancer cells [34]. Nitric oxide donating aspirin is reported to suppress intestinal tumors in *Min* mice and downregulates the expression of PPAR*δ* and enhance apoptosis and perhaps atypical cell death [35]. This suggests that PPAR*δ* contributes to intestinal carcinogenesis.

GW501516 was shown to be a PPAR*δ* subtype-selective ligand using combinatorial chemistry and structure-based drug design [36]. There are some reports describing the effects of PPAR*δ* ligand on colon carcinogenesis. Exposure of *APC* ^min/+^ mice to the GW501516 resulted in activation of PPAR*δ* and significant acceleration of intestinal adenoma growth [37]. Furthermore, PPAR*δ* activation by PPAR*δ* ligand promotes tumor growth by inhibiting epithelial tumor cell apoptosis through activation of a VEGF autocrine signaling loop in *APC* ^min/+^ mice [38]. GW501516 stimulates proliferation of human breast, prostate, and hepatocellular carcinoma cells [39, 40]. In a mouse mammary tumorigenesis model, GW501516 activates 3-phospholinositide-dependent protein kinase-1 that is oncogenic when expressed in mammary ductal cells, and leads to accelerated tumor formation [41]. From these findings, PPAR*δ* selective-ligand tends to exert enhancing effects on carcinogenesis, while its antagonists are expected to prevention and/or treatment of cancer.

## 4. PPAR*γ* LIGANDS AND CARCINOGENESIS

PPAR*γ* plays an important role in the regulation of proliferation and differentiation of several cell types. PPAR*γ* is known to be expressed in various organs, including adipose tissue [42], mammary glands [43], small intestine [44], lung [45], colon [44], and stomach [46], and is also upregulated in various types of cancer cells.

**Figure 2:**
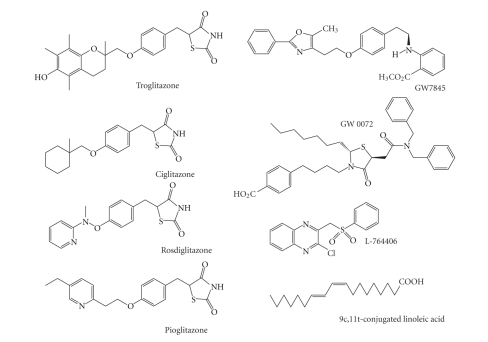
Synthetic and naturally occurring ligands for PPAR*γ*.

This receptor has the ability to bind a variety of small lipophilic compounds derived from both metabolism and nutrition. These ligands, in turn, direct cofactor recruitment to PPAR*γ*, regulating the transcription of genes in a variety of complex metabolic pathways. Several specific ligands (Figure 2) have been identified, such as the thiazolidinediones (including pioglitazone, rosiglitazone, and troglitazone), naturally occurring lipid, polyunsaturated fatty acids (PUFA) (including arachidonic, oleic, and linoleic acid) and the cyclopentenone prostaglandin (PG) 15-deoxy Delta_12,14_-PGJ_2_, a metabolite of PGD_2_. PPAR*γ* ligands have been reported to induce cell differentiation and apoptosis in several types of cancer [47–51], suggesting potential application as anticancer agents. Furthermore, some reports recently suggested that PPAR*γ* ligands can be used as chemopreventive agents for colon, breast, and tongue carcinogenesis [52–54].

**Figure 3:**
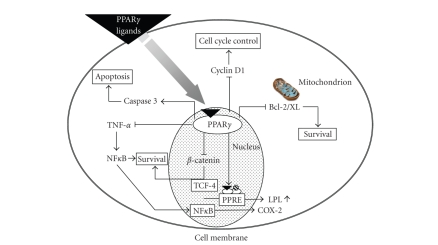
Molecular mechanisms for anticarcinogenic and/or chemopreventive effects of PPAR*γ* ligands.

The most widely used synthetic agents belong to the thiazolidinedione class of antidiabetic drugs (also referred to as glitazones). These include ciglitazone, troglitazone, pioglitazone, rosiglitazone, and LY171.833. Pioglitazone, rosiglitazone, and troglitazone have already been used clinically to treat type 2 diabetes, making use of the ability of synthetic PPAR*γ* ligands to sensitize insulin and to lower blood glucose concentration. Recent evidence indicates that certain thiazolidinedione members, especially troglitazone and ciglitazone, exhibit moderate anitproliferative activities against epithelial-derived human cancer cell lines, including those of prostate [55], breast [56], colon [57], thyroid [51], lung [58], and pituitary carcinoma [50]. PPAR*γ* is known to be expressed in a variety of cancer, and the treatment of these cancer cells with PPAR*γ* ligands often induces cell differentiation and apoptosis [47–51], and exerts antiproliferative effects on human colon cancer [59], breast cancer [47], pituitary adenomas [50], gastric cancer [60], and bladder cancer [61]. Furthermore, postulated mechanisms by which PPAR*γ* ligands exert their effects include modulation of the oncogenic Wnt pathway, inhibition of nuclear factor kappaB (NF-*κ*B), and modulation of cell cycle pro and antiapoptotic proteins (Figure 3). Wnt signaling is a complex pathway in which *β*-*catenin* binds to transcription factors in the nucleus and plays a role as a central mediator in regulating cell proliferation and differentiation [62]. PPAR*γ* activation causes a decrease in *β*-*catenin* expression in adipocytes in vitro and in normal intestinal mucosa in mice [63]. In the cultured human monocytes, PPAR*γ* inhibits NF-*κ*B activation thus influencing the transcription of both survival- and apoptosis-related genes [64]. PPAR*γ* activation also induces the activation of the proapoptotic caspase-3 protein in human liver cancer cell lines and a reduction in antiapoptotic Bcl-2 and Bcl-XL protein level in human colon and gastric cancer cell lines, respectively [65–67]. Furthermore, colon cancer development is related to hyperlipidemia [68], with clear links to high level of serum triglycerides (TGs) [69]. A PPAR*γ* ligand, pioglitazone, suppresses both hyperlipidemia and intestinal polyp formation in the APC-deficient mice in conjunction with elevation of lipoprotein lipase (LPL), which catalyzes TG hydrolysis [70].

We previously investigated the modifying effects of PPAR*γ* or *α* ligands (troglitazone, pioglitazone, or bezafibrate) on early phase of colon carcinogenesis with or without colitis in male F344 rats [19, 71]. The role of PPAR*γ* in AOM-induced colon tumorigenesis was directly demonstrated by the study showing that the incidence of colonic tumors increased in the hemizygous knockout of PPAR*γ* that received AOM [72]. Although thiazolidinediones inhibit AOM-induced colon carcinogenesis in the wild type mice, the observation using APC-deficient mouse models showed conflicting results regarding the effects of PPAR*γ* ligand treatment [73–76]. This may be caused by use of different PPAR*γ* agonists (troglitazone versus pioglitazone), and different doses (100–2000 ppm in diet) examined. Colonic inflammation is associated with a high risk of colorectal cancer (CRC) [77]. CRC is thus one of the most serious complications of inflammatory bowel disease, such as ulcerative colitis and Crohn's disease [77]. In the experiments, dietary administration of PPAR*α* or *γ* ligands effectively suppressed azoxymethane (AOM)-induced or dextran sodium sulfate (DSS)/AOM-induced aberrant crypt foci, which are precursor lesions for colon carcinoma (Table 1). Our findings suggested that synthetic PPAR*γ* and PPAR*α* ligands are able to inhibit the early stages of colon tumorigenesis with or without colitis, and the findings were confirmed by the study conducted by Osawa et al. [78]. Furthermore, we demonstrated ligands for PPAR*γ* and PPAR*α* inhibit colitis-related colon carcinogenesis [79] using our AOM/DSS mouse model [80]. In the experiment, dietary administration (0.05% in diet for 14 weeks) with troglitazone and bezafibrate significantly inhibited both the incidence and multiplicity of colonic adenocarcinoma induced by the treatment with AOM/DSS, although bezafibrate feeding did not significantly lower the multiplicity (Table 2). Dietary exposure of troglitazone and bezafibrate suppressed cell proliferation and induced apoptosis and lowered immnoreactivity of COX-2, inducible nitric oxide, and nitrotyrosine in the colonic malignancies.

PPAR*γ* receptors are activated by certain lipophilic ligands, such as PUFAs and eicosanoid derivatives. They bind to the PPAR*γ* receptor at micromolar concentrations. The essential fatty acids (arachidonic acid, docosahexanoic acid, and eicosapentaenoic acid) as well as modified oxidized lipids (9-hydroxyoctadecanoic acid and 13-hydroxy-octadecanoic acid) bind to and activate PPAR*γ* [5]. Recently, conjugated linoleic acid (CLA) was shown to act as a high affinity ligand and an activator of PPAR*γ* [81]. Anticarcinogenic activity of CLA is mediated by PPAR*γ* activation in susceptible tumors [81]. When treated with CLA, PPAR*γ* expression is increased, and APC and c-myc proteins are downregulated in the human colon cancer cells, and finally proliferation of cancer cells is inhibited by CLA [82–85]. In fact, feeding with seed oils containing 9*c*, 11*t*, 13*t*-, 9*c*, 11*t*, 13*c*-, and 9*t*, 11*t*, 13*c*-conjugated linolenic acid, which are converted to 9*c*, 11*t*- and 9*t*, 11*t*-CLA within colonic and liver cells, suppresses AOM-induced colon carcinogenesis by increased expression of PPAR*γ* protein in the colon mucosa [86–89].

## 5. CLINICAL TRIAL FOR PPAR*γ* LIGANDS AGAINST TUMORS

There are several clinical studies on the effects of PPAR*γ* ligands on malignancies (Table 3). The beneficial effects of glitazones on liposarcomas have been demonstrated in a small clinical trial [90]. Three patients with intermediate to high-grade liposarcomas were given troglitazone (800 mg/day orally). In the patients, differentiation of the neoplasms occurred as revealed by histological and biochemical analysis. The clinical outcome of these patients was not reported, but the therapy was well tolarated [90]. However, a phase II study on 12 patients with liposarcoma showed that the PPAR*γ* ligand rosiglitazone did not significantly improve clinical outcome [94]. In prostate, PPAR*γ* immunoreactivity was significantly higher in prostate cancer and prostatic intraepithelial neoplasia than in those with benign prostate hyperplasia and with healthy prostate [98]. A high incidence of prolonged stabilization of serum prostate-specific antigen (PSA) was observed in phase II clinical study, where patients with advances prostate cancer who had no symptoms of metastasis were treated with troglitazone (800 mg/day orally). Moreover, one patient had a striking decrease in PSA concentration to almost undetectable amounts [91]. In a 75-year-old man with occult recurrent prostate cancer showed a decrease in PSA after oral treatment with toroglitazone (600–800 mg/day for 1.5 years) [92]. Thus, PPAR*γ* is expressed in prostate cancer and activation of PPAR*γ* might offer an additional therapeutic option for treatment of prostate cancer in the near future. At present, most of the available data suggest that PPAR*γ* has antineoplastic effect on malignant neoplasms [99], including colonic malignancies. However, in a clinical phase II study on CRC, orally administrated troglitazone did not lengthen median progression-free survival or median survival in 25 patients with chemotherapy-resistant metastatic colon carcinoma [93]. In a phase II study [95] for the use of troglitazone to treat patients with advanced refractory breast cancer, no objective tumor response was observed. However, the study was incomplete because troglitazone was withdrawn from commercial availability after a warning by the US Food and Drug Administration about hepatic toxic effects. On the other hand, it is important to note that neither hormone status of the tumors nor the amount of PPAR*γ* protein is assessed before patients were included in the study. In an open labeled phase II study where ten patients with thyroglobulin-positive and radioiodine-negative differentiated thyroid cancer were enrolled and they were given oral rosiglitazone treatment (4 mg/day for 1 week, then 8 mg per day for 7 weeks), rosiglitazone treatment resulted in a 40% partial response rate, but no complete responses, and the expression level of PPAR*γ* mRNA and protein in the neoplasm appeared unrelated to rosiglitazone treatment response [96]. The findings also suggest that higher doses and longer duration of rosiglitazone therapy may be useful to better define the role of rosiglitazone as a redifferentiation agent in differentiated thyroid cancer. There is a phase I clinical study of a PPAR*γ* ligand (LY293111) that is not thiazolidinedione members [97]. LY293111 is a novel diaryl ether carboxylic acid derivative and is known as PPAR*γ* agonist and LTB4 antagonist. The study suggested the dose (600 mg) of LY293111 in combination with irinotecan (200 mg/m^2^ IV every 21 days for phase II clinical study against solid tumors.

## 6. CONCLUSIONS

PPARs were originally recognized to be genetic regulators of complex pathways of mammalian metabolism, including fatty acid oxidation and lipogenesis. However, the receptors have been shown to be implicated in carcinogenesis and inflammation. PPARs are involved in cell proliferation and differentiation of a variety of cancer. Numerous reports indicate that PPARs ligands could play an important role in prevention and inhibition of cancer development. Synthetic PPAR ligands used for drugs or those of naturally occurring lipids are promising cancer chemopreventive agents with slight side effects against several types of cancer. We should characterize expression patterns of different isoforms of PPAR in cancerous and precancerous tissues and determine their precise roles in the carcinogenic process for development of PPARs ligands as a novel class of cancer preventive/theraputic drugs. Based on current data from preclinical and clinical studies, we believe that thiazolidinediones, especially PPAR*γ* agonists, have important role in short-term prophylactic therapy designed to reduce the number of putative preneoplasia, ACF, in patients who are at high risk for CRC development.

## Figures and Tables

**Table 1:** Effects of PPAR ligands on ACF formation in rats.

Treatment (No. of mice)	ACF/colon (% inhibition)	ACs/colon (% inhibition)
AOM alone (12)	83 ± 6^(a)^	2.0 ± 0.24
AOM + 0.01% troglitazone(8)	68 ± 16 (18%)	1.7 ± 0.21(15%)
AOM + 0.05% troglitazone(8)	55 ± 13^(b)^ (34%)	1.5 ± 0.13^(c)^ (25%)
AOM + 0.01% bezafibrate (8)	75±8 (10%)	2.0 ± 0.20 (0%)
AOM + 0.05% bezafibrate (8)	53± 9^(d)^ (36%)	1.9 ± 0.10(5%)
None	0	0

1% DSS + AOM (10)	115 ± 22	2.4 ± 0.29
1% DSS + AOM + 0.01% pioglitazone (7)	71 ± 24^(e)^ (38%)	1.8 ± 0.17^(f)^ (25%)
1% DSS + AOM + 0.01% troglitazone(7)	57 ± 14^(g)^ (50%)	1.6 ± 0.14^(g)^ (33%)
1% DSS + AOM + 0.01% bezafibrate (7)	59 ± 18^(h)^ (49%)	1.7 ± 0.16^(i)^ (29%)
None	0	0

^(a)^Mean ±SD. ^(b–d)^Significantly different from the AOM alone group: ^(b)^ *P* < .01; ^(c)^ *P* < .005; and ^(d)^ *P* < .001. ^(e–i)^Significantly different from the DSS/AOM group: ^(e)^ *P* < .05; ^(f)^ *P* < .01; ^(g)^ *P* < .001; ^(h)^ *P* < .005; and ^(i)^ *P* < .002.

**Table 2:** Effects of PPAR ligands on colon carcinogenesis in mice.

Treatment (no. of mice)	Incidence/Multiplicity (% inhibition)		
	Total	Adenoma	Adenocarcinoma
AOM/DSS	100%/5.2 ± 3.0^(a)^	100%/2.1 ± 1.8	100%/3.0 ± 1.8
AOM/DSS/0.05% Troglitazone	90%/2.5 ± 1.8^(b)^(52%,)	90%/1.6 ± 1.1 (24%)	40%^(c)^/1.2 ± 2.5^(b)^ (60%)
AOM/DSS/0.05% Bezafibrate	80%/2.6 ± 2.5^(b)^(50%)	70%/1.1 ± 1.0^(b)^ (48%)	60%^(b)^/1.8 ± 2.6 (40%)
None	0%/0	0%/0	0%/0

^(a)^Mean ±SD. ^(b,c)^Significantly different from the AOM/DSS group: ^(b)^ *P* < .05; and ^(c)^ *P* < .01.

**Table 3:** Clinical trials on the anticancer effects of PPAR*γ* ligands.

Clinical trials	Drug	Results	Reference no.
Patients with intermediate to high-grade liposarcomas (case reports)	Troglitazone	Histlogical and biochemical differentiation	[90]
Phase II study on patients with histologically-confirmed prostate cancer and no symptomatic metastatic disease	Troglitazone	Lengthened stabilization of prostate-specific antigen	[91]
75-year-old patient with an occult recurrent prostate cancer (case reports)	Troglitazone	Reduced prostate-specific antigen	[92]
Phase II study on patients with metastatic colon cancer	Troglitazone	No significant effect	[93]
Phase II study on patients with liposarcoma	Rosiglitazone	Lengthened mean time of progression	[94]
Phase II study on patients with refractory breast cancer	Troglitazone	No significant effect	[95]
Phase II study on patients with thyroglobulin-positive and radioiodine-negative differentiated thyroid cancer	Rosiglitazone	Induced radioiodine uptake	[96]
Phase I study on patients with solid tumors	LY293111	The recommended oral dose (600 mg/day) for phase II trial	[97]

## ABBREVIATIONS

AOM: Azoxymethane
APC: Adenomatous polyposis coli
CLA: Conjugated linoleic acid
COX-2: Cyclooxygenase-2
CRC: Colorectal cancer
DSS: Dextran sodium sulfate
LPL: Lipoprotein lipase
LTB4: Leukotriene B4
PG: Prostaglandin
PPARs: Peroxisome proliferators-activated receptor
PPRE: Peroxisome proliferators response element
PSA: Prostate-specific antigen
PUFA: Polyunsaturated fatty acid
RXR: Retinoid X receptor
TG: Triglyceride
